# Toward “Safe” Chemicals and Materials on Mars: Knowledge Gaps for Expanding Planetary Protection Requirements

**DOI:** 10.1021/acs.est.5c15572

**Published:** 2026-03-27

**Authors:** John D. Hader, Alberto G. Fairén, Marlene Ågerstrand, Matthew MacLeod, Bernd Nowack

**Affiliations:** † 111825 Swiss Federal Laboratories for Materials Science and Technology (Empa), Technology and Society Laboratory, St. Gallen 9014, Switzerland; ‡ Centro de Astrobiología (CAB), CSIC-INTA, Madrid 28850, Spain; § Department of Astronomy, Cornell University, Ithaca, New York 14853, United States; ∥ Department of Environmental Science, 7675 Stockholm University, Stockholm 106 91, Sweden

**Keywords:** safe and sustainable by design, chemicals management, planetary protection, chemical fate and transport, microplastics, chemical regulation, multilateral environmental agreements

## Abstract

The United Nations Outer Space Treaty states that the exploration of celestial bodies must avoid “harmful contamination” which may impede scientific exploration by other parties to the treaty. To guide treaty compliance, Planetary Protection regulations promulgated by the Committee on Space Research set limits for microbial contamination of celestial bodies, particularly those that may harbor extant life (e.g., Mars). However, anthropogenically introduced chemicals and materials are not regulated but may cause “harmful contamination” and thus pose a potential threat to scientific exploration. On Earth, threats from anthropogenic chemicals and materials are often managed by considering both potential exposure to the substances and their hazardous properties. The lack of knowledge around hazards to possible extant life on Mars means that chemicals and materials should be designed and used so that their exposure concentrations are minimized. Here, we review possible emission, partitioning, persistence, and transport processes on Mars for anthropogenically introduced chemicals and materials and identify key knowledge gaps. We highlight difficulties and lessons learned from pollution policy development on Earth that could inform interplanetary chemical and material management. This work aims to support the expansion of the Planetary Protection guidelines to include a “No- or Low-Exposure by Design” approach to chemicals and materials on Mars.

## Graphical abstract

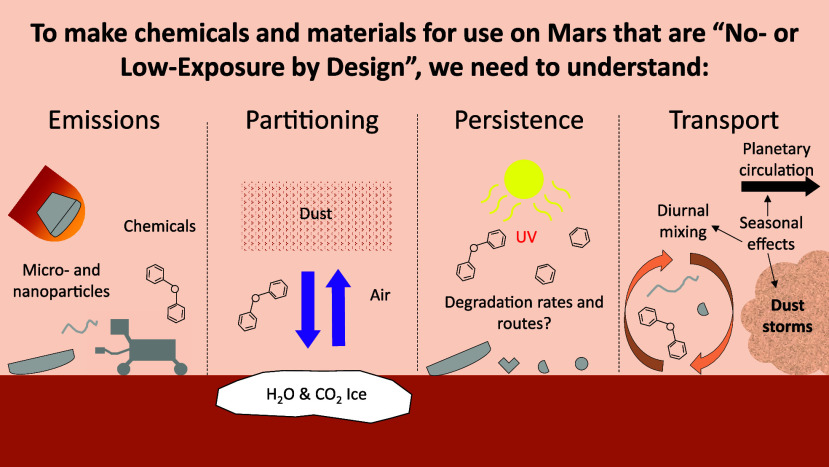

## 1 Introduction

Several national governments and private companies have announced plans to send humans to the surface of Mars in the coming decades.^1–5^ Such exploratory missions come with the unavoidable release of chemicals and materials from Earth into the Martian environment, as evidenced by photographs of debris contaminating the Martian surface from several uncrewed missions over the past decades (see ref 6 and Figure 1). The United Nations Outer Space Treaty (OST), signed in 1967 and now having 118 parties,^7^ requires that countries conduct exploration of celestial bodies (i.e., Earth’s moon, planets, the moons of other planets, and other solar system objects like asteroids and comets) without causing “harmful contamination” (Article IX).^8^ However, the exact nature of “harm” or “contamination” covered by this clause of the OST was not explicitly defined. Instead, the Committee on Space Research (COSPAR) Panel on Planetary Protection is tasked with developing and promulgating nonbinding guidelines for parties to the OST to avoid “harmful contamination” of celestial bodies.^9,10^ To date, the COSPAR guidelines focus only on restricting *microbial* contamination resulting from human exploration, as the introduction of microbes or biologically relevant organic chemicals from Earth would threaten the scientific investigation of signs of past or present life on celestial bodies.

**Figure 1:**
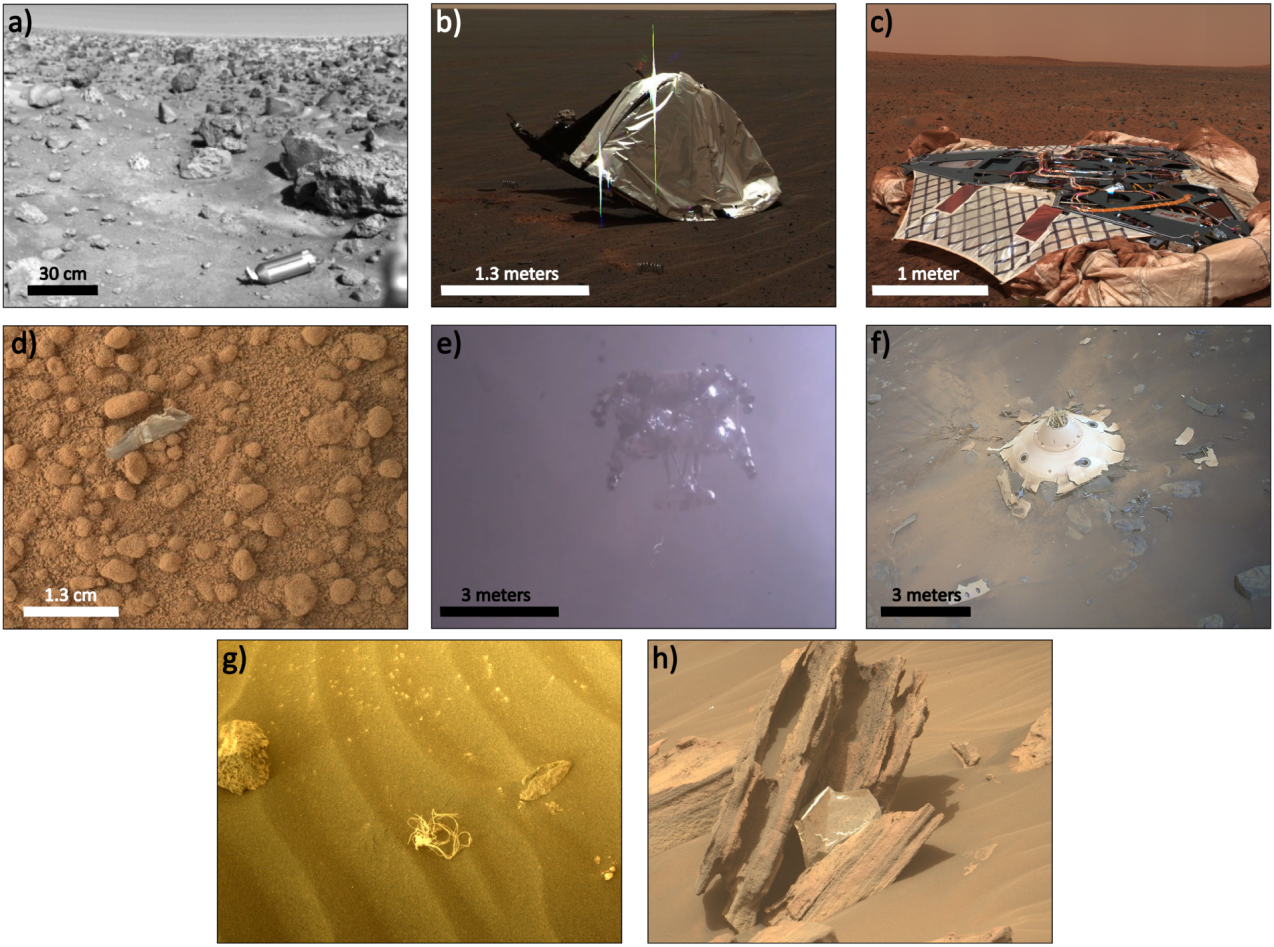
Images of anthropogenically introduced materials on or near the surface of Mars. a) Surface sampler collector head aluminum shroud ejected from the Viking 2 lander (1976). b) Heat shield debris from the Opportunity rover (2004). c) Landing platform of the Spirit rover (2004). d) Likely debris from the Curiosity rover (2012). e) Skycrane lowering the Perseverance rover to the Martian surface, prior to its intentional crash landing at a safe distance (2021). f) Perseverance rover’s entry, descent, and landing backshell, imaged by the Ingenuity helicopter (2022). g) String-like material debris, likely from the Perseverance rover entry, descent, and landing stages (2022). h) A piece of multilayer insulation caught on a Martian rock, likely blown in the wind from the Perseverance rover Skycrane explosion 2 km away (2022). See ref 11 for details regarding panels b, d, e, f, g, and h. Image credits: a) ref 12, b) ref 13, c) ref 14, d) ref 15, e)­ref 16, f)­ref 17, g)­ref 18, h) ref 19. See also ref 20 for an image of China’s Zhurong rover landing platform from 2021.

The production, use, and disposal of anthropogenic chemicals and materials on Earth has resulted in many unintended negative consequences in Earth’s biotic and abiotic systems throughout the 20th and 21st centuries. Chemicals in commerce have accumulated in humans and other biota, causing negative health and environmental outcomes. Examples include per- and polyfluoroalkyl substances (PFAS),^21,22^ polychlorinated biphenyls (PCBs),^23^ and dichlorodiphenyltrichloroethane (DDT) and other pesticides.^24,25^ Chemicals such as chlorofluorocarbons (CFCs) have also accumulated unexpectedly in Earth’s stratosphere, causing damage to the ozone layer.^26^ Furthermore, micro- and nanoparticles from anthropogenic plastics and other materials have been released into environmental media everywhere in the world, possibly affecting environmental and human health.^27,28^ Given the impacts of pollution on Earth, anthropogenically introduced chemicals and materials associated with robotic or crewed exploration missions could also cause “harmful contamination” to a celestial body, either to undiscovered life (if it exists)^29,30^ or to poorly understood abiotic processes.^6,31^

In light of the issues caused by chemical and material pollution on Earth, and the growing environmental footprint of humanity in off-Earth environments, Hader et al. (2023) called for the COSPAR Planetary Protection guidelines to be revised.^6^ They highlighted the need for guidelines to address anthropogenically introduced chemical and material contamination, supported by further research to determine the chemical and material engineering requirements for avoiding “harmful contamination” and enabling the sustainable exploration of celestial bodies. Given the high level of international interest in sending humans to Mars in the coming decades, and the fact that chemicals and materials for use by humans on Mars are currently being designed,^32^ developing an understanding of how to avoid *harmful* chemical and material contamination on Mars is a particularly pressing issue.

The “Safe and Sustainable by Design” (SSbD) approach is a policy framework that builds on “Green Chemistry” principles formalized in the 1990s^33^ to inform how chemicals and materials can be developed, produced, used, and disposed of in a manner that is safe and sustainable throughout their lifecycles.^34,35^ While the Green Chemistry and SSbD approaches are the standard for environmentally responsible chemical and material development on Earth, these approaches are not directly applicable to the development and use of chemicals and materials on Mars in the context of avoiding the “harmful contamination” outlawed by the Outer Space Treaty. Specifically, the aspect of assessing and designing for “Sustainability” within the SSbD approach utilizes Life Cycle Assessment (LCA) to determine how to minimize the environmental footprint of the feedstocks, production, use stage, and end of life of chemicals and materials, assessing, e.g., the climate change impacts or resource use attributable to the chemicals/materials across their life cycle. While this question of environmentally sustainably producing and using chemicals/materials on Mars would be relevant in the context of assessing the sustainability of in situ resource utilization (i.e., the process of using resources at a celestial body to produce chemicals/materials there, as opposed to bringing them from Earth),^36^ answering these larger-scale sustainability questions around production in the Martian environment extends beyond the scope of whether anthropogenically introduced chemicals/materials cause “harmful contamination”, and thus is not within the scope of this study (though readers are referred to ref 31 for some discussion around this topic).

However, the aspect of “Safety” defined by the SSbD approach is relevant in the context of avoiding “harmful contamination” on Mars, as this aims to assess and minimize through design choices “...the potential risks posed by chemicals and materials to human health and the environment throughout the life cycle...”^35^ A key aspect, though, regarding how this safety consideration is applied on Earth limits its applicability to protecting the Martian environment. On Earth, “safe” chemicals and materials are those that do not cause adverse outcomes in humans or the environment (i.e., through direct or indirect biological effects or ozone depletion). A risk-based approach to safety assesses whether a chemical or material poses a hazard to living organisms (e.g., toxic effects to reproduction, mutagenic effects, or carcinogenic effects)^37^ and whether (or by how much) the expected exposure in a given medium exceeds the concentration where the adverse outcome occurs (i.e., risk = exposure concentration/hazardous effect concentration).^34^ In a hazard-based approach to safety, chemicals are managed based on their hazardous properties only, regardless of expected exposure. Given our limited understanding of the Martian environment, any potential chemical or material hazards to biota (if they exist) can only be speculated about, as can the potential effects on the abiotic environment. Therefore, the most feasible approach to “safe” chemicals and materials use on Mars is to reduce environmental concentrations of these chemicals and materials as much as possible, since lower exposure in any media would pose a lower risk to any possible extant life or abiotic processes.

Thus, given our limited knowledge of the Martian environment, a “No- or Low-Exposure by Design” framework would be a precautionary approach to the management of chemicals and materials on Mars for avoiding “harmful contamination”, and would be a more fit-for-purpose framework than a “Safe and Sustainable by Design” approach, given the fundamental limitations associated with attempting to protect an environment for which we only have incipient knowledge. We propose the construct of a “No- or Low-exposure” by design approach—as opposed to simply a “No exposure by design” approach—as a recognition of the fact that most, if not all, activities humans conduct in the Martian environment will result in emissions of at least some anthropogenically introduced chemicals or materials (i.e., “No exposure” is not feasible), but that such activities should be designed in a way that as low exposure as possible occurs in the Martian environment. However, the development of such an approach requires understanding how anthropogenically introduced contamination behaves in the Martian environment. To support expanded Planetary Protection guidelines and to help move toward a framework of “No- or Low-Exposure by Design” of chemicals and materials on Mars, the aims of this study are to

1. Collect an inventory of the mass of anthropogenic chemicals and materials that have already been sent to the Martian environment, and gain insight into the types of chemicals and materials that may be used in exploration missions in the coming decades.
2. Review environmental processes that determine chemical and material emissions, partitioning, persistence, and transport on Mars.
3. Identify key data collection and modeling studies needed to close knowledge gaps and enable revised Planetary Protection requirements based on a “No- or Low-Exposure by Design” approach.
4. Identify and discuss some of the challenges and key lessons learned regarding the development of international chemical and material management policies, which could inform how to implement interplanetary chemical and material management.

We underscore that the end points of interest for protection against exposure to anthropogenically introduced chemicals and materials in the Martian environment are extant Martian life (if it exists) and potentially sensitive abiotic processes, whose disruption by chemicals and materials could result in the “harm” that the Outer Space Treaty and Planetary Protection guidelines aim to protect against. We leveraged the collective knowledge of the authors regarding chemical and material contamination processes on Earth and known processes in the Martian environment as the starting point for surveying relevant literature on the topics covered and for selecting illustrative examples of relevant studies based on expert judgment. While this enabled the broad scope covered in this study, systematic literature reviews of key elements related to anthropogenic contamination of the Martian environment are left for future work and for filling the identified knowledge gaps (see Section 4).

## 2 Understanding Anthropogenic Chemicals and Materials on Mars

As of February 2026, 17 uncrewed exploration missions have entered the Martian environment, beginning with the Russian Mars-2 lander, which crash-landed in 1971, and most recently with the Chinese Zhurong rover which successfully landed in 2021 (see Table S1). In total, five missions crashed due to either failure of the entry, descent, and landing sequence upon arrival (Mars 2, Mars 6, Mars Polar Lander, and the ExoMars Schiaparelli) or accidental entry of the spacecraft into the atmosphere while attempting to enter orbit (Mars Climate Orbiter). Ten missions successfully landed but are now defunct, and two missions (NASA’s Curiosity and Perseverance rovers) are still operational. From these vehicles, a total of roughly 20,000 kg of anthropogenically introduced chemicals and materials have been sent to the Martian environment, either to the surface or into the atmosphere. Figure 2 displays a map of the Martian surface, overlaid with the approximate locations of all the exploratory missions that arrived at the surface of Mars. It demonstrates that locations with anthropogenically introduced chemicals and materials are distributed widely across both the northern and southern hemispheres. Furthermore, over 23,000 kg of materials remain or are thought to remain in Martian orbit from nine defunct and seven operational satellites. While orbital information is not available for all defunct satellites, at least two of these satellites (the Mariner 9 and Viking 1 orbiters) were in orbits that may have caused the spacecraft to fall into the Martian atmosphere in recent years (see Table S1).

**Figure 2:**
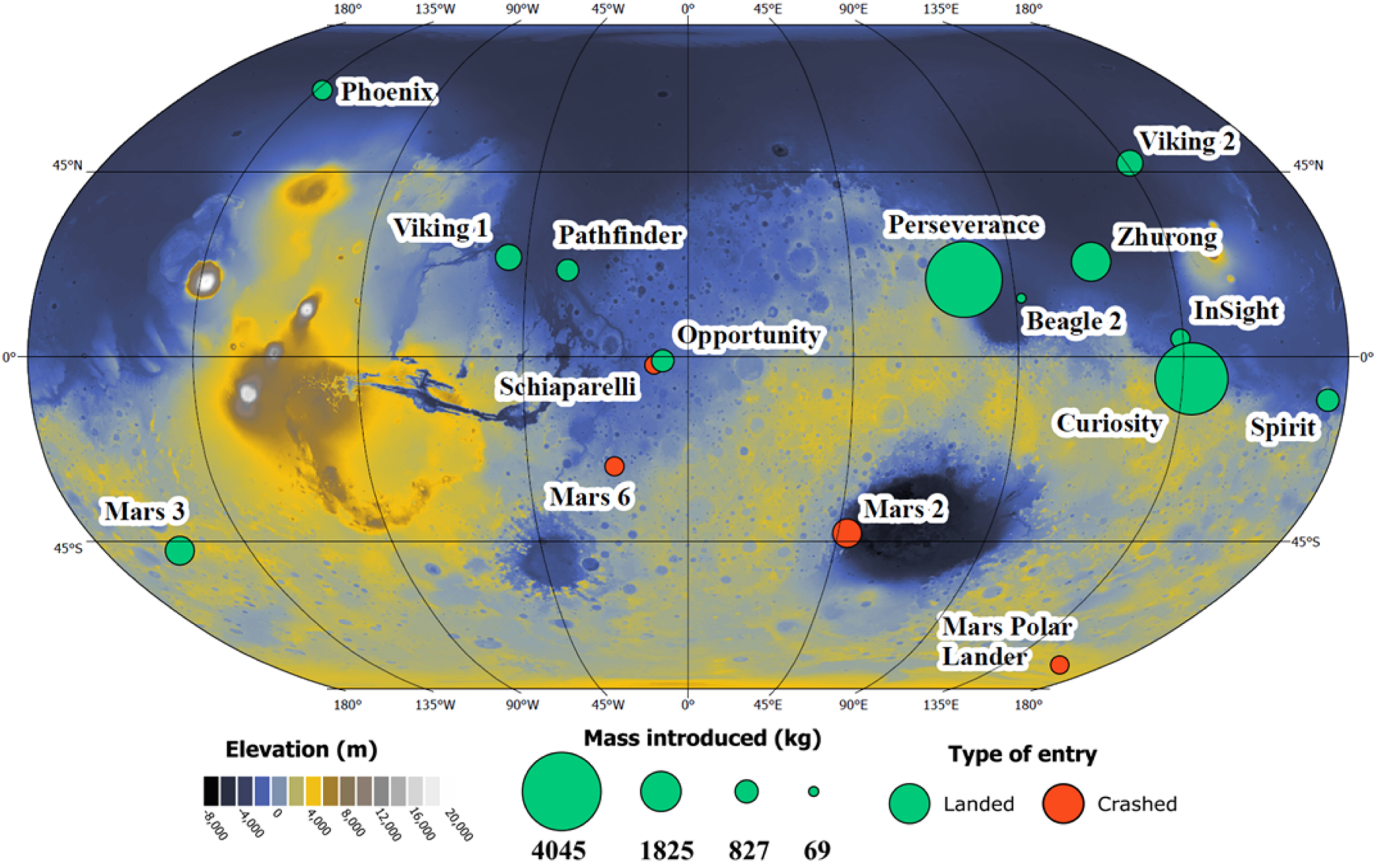
Elevation map of Mars, with approximate locations of uncrewed missions that delivered anthropogenic chemicals and materials into the Martian environment. The size of circles roughly corresponds to the mass introduced. Not shown is roughly 338 kg introduced by the Mars Climate Orbiter, which inadvertently plunged into the atmosphere when attempting to enter orbit due to commands being sent to the satellite in English instead of metric units.^38^ Missions list and approximate locations on Mars obtained from refs 39 and 40. See Table S1 for more details. Elevation data from ref 41. Map generated using ArcGIS Pro (Version 3.4; Esri).

Regarding the types of chemicals and materials used in robotic missions to the surface of Mars, COSPAR Planetary Protection guidelines require that an inventory of the individual organic materials in a spacecraft be made for those materials used at a mass over 1 kg,^9^ with NASA further requiring organic materials present in masses greater than 25 kg to also have samples of the materials archived.^42^ The purpose of these inventories is to provide a cross-check for organic chemicals that might be measured at the surface of Mars and enable validation of whether the measured organic chemicals were native in situ compounds and not chemicals from spacecraft contamination.^42–44^ However, the specific identity of some of these chemicals and materials that compose the organic inventories and archives is proprietary information, limiting access by the scientific community.^45^ Information on such constituents of spacecraft sent to the surface of Mars is also lacking from China’s recent Zhurong rover.^46^ Thus, increased transparency surrounding the chemicals and materials sent to Mars is a key data need to enable independent and transparent assessment of the contamination potential.

Despite this limitation, some general information regarding the chemicals and materials utilized in previous spacecraft exists in the public domain. Debus (2005) reports that various iron, aluminum, and titanium alloys can be used, that silicon-based materials may be present in optics and electronics, that plutonium­(IV) oxide may be used as a radioisotope for power generation, and that various metals may be present in electronics and/or batteries, such as nickel, lithium, and tin–lead and copper-based alloys.^47^ Regarding organic materials, items such as parachutes, airbags, thermal covers, coatings, paints, and wires can contain polyester, polyamide, polyimide, polyurethane, and polytetrafluoroethylene (PTFE, or Teflon) polymers, as well as various other unknown additional chemicals like UV stabilizers and pigments.^47,48^ Similarly, NASA has published a template for an organic material inventory for mission Planetary Protection compliance that provides examples of different adhesives, primers, paints, lubricants, inks, plastics, and other organic-containing materials that may be used in exploratory missions.^42^ Lakdawalla (2018) also provides details about the construction and components of the Curiosity rover, noting chemicals and materials like polyester, nylon, Technora, and Kevlar being used in the parachute, Teflon being present in the rock drilling apparatus, organic check materials containing a “fluorinated hydrocarbon chemical”, and various organic solvents in the onboard gas chromatography–mass spectrometry instrument.^49^ Hydrazine (N_2_H_4_) is a common fuel used for retrorocket burns during descent stages of vehicles, and a common heat shield material is NASA’s phenolic-impregnated carbon ablator.^50,51^ Fractions of organic versus inorganic or metallic amounts of the materials and chemicals present in spacecraft are difficult to ascertain due to a lack of transparent information on spacecraft contents. However, it is likely that more inorganic/metallic material would be present than organic materials, given that fewer than 12 materials typically reach the >25 kg requirement for reporting in NASA’s organic materials archive.^48^

While crewed missions to Mars are still in the planning phase, the chemicals and materials that will be used in the support infrastructure for surface habitats and operational equipment are currently being researched and developed. For example, samples of possible materials for use in extravehicular activity suits were included on the outside of the Mars Perseverance rover to investigate how their chemical composition changes from exposure to in situ Martian conditions. These samples included Dacron (a type of polyester), Vectran (produced from liquid crystal polymer), and Teflon (a PFAS-containing material).^52^ Additionally, various polymer-based aerogels for radiation and thermal protection are under development for potential use in a Mars exploration mission.^53^

While the limited information regarding the detailed composition of materials sent to the Martian environment can be used as a starting point for discussing the processes of contaminant emissions, fate, and transport (see Section 3), this is an area that should be closely monitored and updated as the technology used for sending crewed and uncrewed missions to Mars develops and expands. Furthermore, the need for increased transparency regarding chemicals and materials sent to Mars is discussed in Section 4.

## 3 Review of Chemical and Material Fate and Transport Processes on Mars

In discussing the potential interactions between environmental processes on Mars and anthropogenically introduced contamination in the form of chemicals and materials, we focus on the following processes: 1) emissions of chemicals and micro- and nanoparticles from materials; 2) partitioning of chemicals between different environmental media; 3) persistence of contamination; and 4) contaminant transport processes. These processes are summarized in Figure 3.

**Figure 3:**
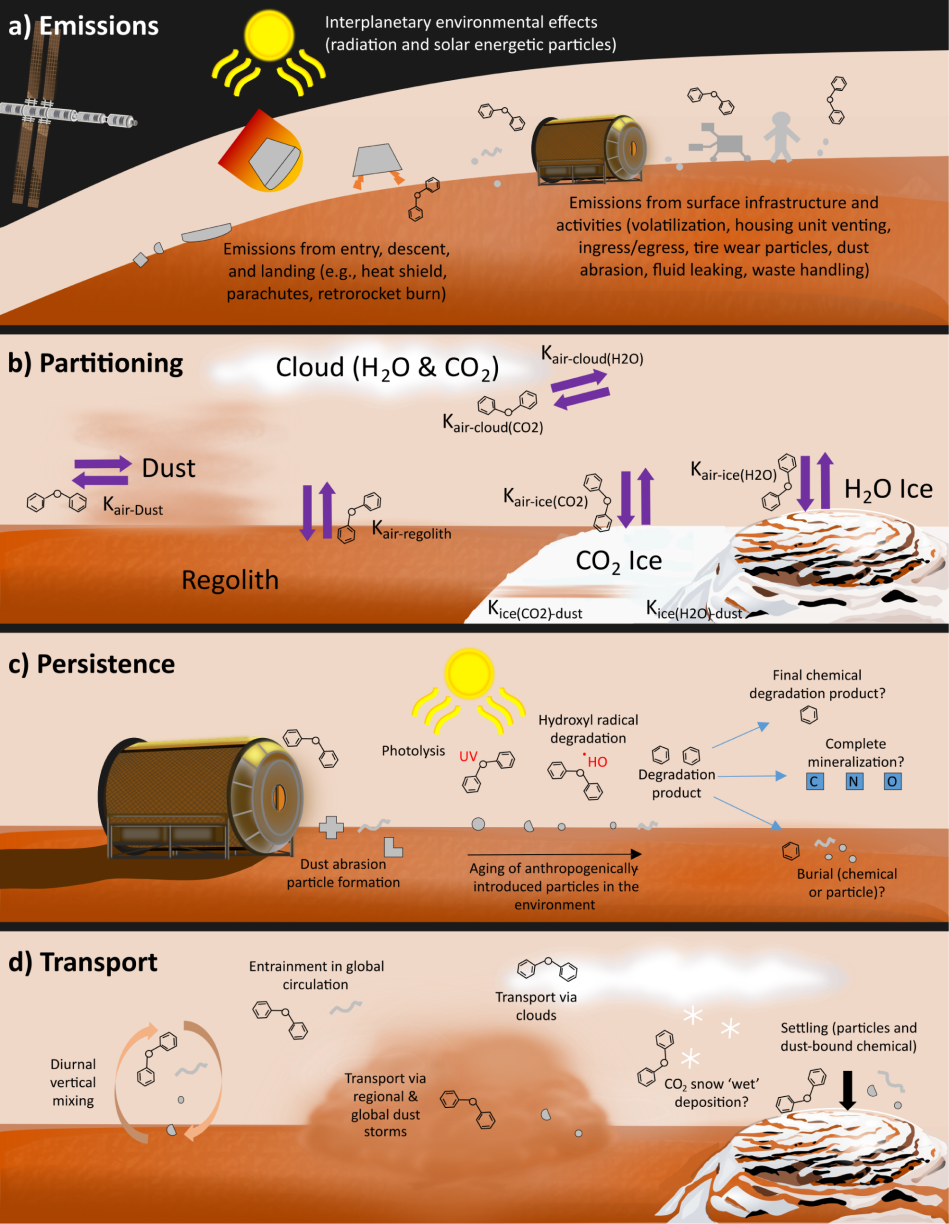
Illustration of processes that may affect anthropogenically introduced chemicals and materials throughout their life cycle during and after an exploratory mission to Mars. Distances and sizes are not to scale. Figure generated in part with Inkscape Versions 1.3.2 and 1.4. Background artwork by Marissa Kosnik.

### 3.1 Emissions of Chemicals and Micro- and Nanoparticles

While there are many unknowns associated with how chemicals and material micro- and nanoparticles (henceforth “particles”) may be emitted from human exploration missions on Mars, emission mechanisms can be hypothesized based on existing knowledge of chemical and particle emissions on Earth and knowledge from previous missions to Mars (see Figure 3a). Prior to entering the Martian environment, items associated with human exploration will undergo transit in deep space between the Earth and Mars for approximately nine months.^54^ During this time, outside of Earth’s magnetic field, the items will be exposed to galactic cosmic rays and solar energetic particles, with such ionizing radiation having the capacity to alter materials at the molecular level.^55–57^ It has been shown that polymeric material exposure to deep-space radiation can result in significant changes in some macro properties (e.g., tensile strength or ballistic performance), likely owing to chain scission or cross-linking of molecules within the materials.^54^ Once on the Martian surface, materials will be exposed to radiation levels higher than on Earth but lower than in deep-space transit (similar to those on the International Space Station).^58^ Additional research is needed to understand how items exposed to deep-space radiation and radiation on Mars could influence chemical or particle contamination while in use in the Martian environment. As a related example, Harrad et al. (2023) hypothesize that accelerated material aging via ionizing radiation can generate micro- and nanoparticles on the International Space Station.^59^

Emissions of chemicals and materials into the Martian environment could begin during a spacecraft’s entry, descent, and landing phase. Interaction between the spacecraft’s heat shield and the atmosphere upon atmospheric entry would cause pyrolysis of some of the material in the heat shield, but other byproducts from incomplete combustion could also be emitted into the environment.^47,60^ Depending on the dust environment at the time of entry, erosion of the heat shield due to impinging dust particles can also result in emissions of pieces of the heat shield into the atmosphere.^61^ Parachutes are used to slow landing vehicles to subsonic speeds, which, along with their heat shield and connective back shells, are subsequently jettisoned into the environment prior to the final landing sequence (Lakdawalla, 2018; see also Figure 1b and f).^49^ Hydrazine-based retrorocket propulsion used to slow the spacecraft for its descent to the surface (e.g., as was used by the Perseverance and Curiosity rovers) involves emissions of N_2_, H_2_, NH_3_, and carbazic acid.^49,62–64^ Retro-rocket propulsion near the surface can also accelerate Martian surface particles such as dust or sand, which can impinge upon the coatings of the landed mass, causing erosion and emissions of, for example, paint particles.^65^ Such retro-rocket propulsion systems, as well as the inflatable “airbags” that were used for e.g., the final descent stage of the Spirit rover, are subsequently deposited into the Martian landscape upon landing (see Figure 1c and e). The practice employed with the Curiosity and Perseverance “skycrane” of flying this final descent stage to a safe distance from the rover for it to intentionally crash-land also likely caused the emission of large amounts of chemical and material debris (see Figure 1).^11,49^ Additionally, spacecraft in Martian orbit (e.g., refs 66 and 67) that are deorbited at the end of their operational lives could release various metals into the atmosphere—a practice in Earth orbit which poses a potential and growing risk to the atmosphere.^68,69^

Once on the Martian surface, chemicals and materials may be released from the deposited entry, descent, and landing stages (see Figure 1), housing units during their decompression or venting of air, or leaks of fluids from housing units or surface equipment.^6,47,70^ Particle formation could occur through wear on vehicle wheels on the Martian regolith^49^ or from the footwear of the protective suits worn by astronauts. Chemicals could also volatilize from items on the surface,^44,71^ and abrasion of anthropogenically introduced materials by the harsh dust environment may generate particles.^6^ Furthermore, handling any waste produced by humans in the course of their activities could result in emissions of chemicals or material particles if not perfectly contained, stored, and/or removed from the surface after the completion of activities.^72^

While materials for use on Mars would be designed to perform for the necessary lifetime,^32,54^ to the best of the authors’ knowledge, the effect of the Martian environmental conditions (e.g., increased UV radiation, extreme temperatures, and interaction with dust) on the characteristics of any particles formed from the materials and the subsequent impacts on their environmental contamination is not considered in the design of materials for use on Mars. Since the shape, size, and other physical characteristics of microparticle contamination are key in determining their behavior in the environment,^73^ understanding these anthropogenically introduced particle formation dynamics in the Martian environment is key for contamination prevention and management.

### 3.2 Environmental Partitioning of Chemicals

On the Martian surface, chemicals emitted into the environment by human activities may partition between air, regolith, dust (surface or airborne), water-ice cloud particles, CO_2_ ice-cloud particles, surface water ice, and surface CO_2_ ice (see Figure 3b). The partitioning behavior of a chemical can be characterized by theoretically, experimentally, or computationally determining partition ratios of a chemical between two media at equilibrium conditions (e.g., the air–water partition ratio, *K*_aw_).^74^ Partition ratios, however, are highly dependent on temperature. A partition ratio for a chemical between two media determined at given environmental conditions (typically standard temperature and pressure on Earth; 25 °C and 1 bar) can be adjusted to reflect the partition ratio at lower or higher temperatures using the van’t Hoff equation (see eqs 3–51 of ref 74). The temperature dependence of chemical partition ratios is generally investigated in the context of common environmental conditions on Earth (e.g., −5 to 40 °C),^75,76^ though some research reports values down to −30 °C.^77^

It is unclear whether the existing experimental, computational, and theoretical frameworks for the determination of chemical partition ratios could be applied to Martian conditions. For example, the commonly used form of the van’t Hoff equation assumes that the molar volumes of the two media—themselves affected by temperature—remain constant over the temperature range of interest, since there is less than a 10% change in molar volumes for temperatures between 0 and 30 °C.^74^ Additionally, the van’t Hoff equation is based on the assumption that the enthalpy change (Δ*H*) of the compound between the two media is constant over small temperature ranges.^74^ Extrapolating a partition ratio determined at a standard Earth temperature of 25 °C to a common nighttime temperature on Mars of −80 °C (see ref 78) could therefore introduce large errors in partition ratios, as the robustness of these assumptions underlying the van’t Hoff equation may no longer hold satisfactorily. Furthermore, the extreme spatial and temporal environmental changes during the Martian day would present unique chemical fate modeling challenges not seen on Earth. As observed by the Perseverance Rover (located roughly 18° north of the equator), temperatures on and near the Martian surface just before sunrise can approach −80 °C. By local solar noon, surface temperatures can be as high as −3 °C, while temperatures 1.5 m above the surface are still below −30 °C.^78^ With such large temperature differences, the partition ratios of chemicals, as well as the phase the chemical would naturally be present in (i.e., gas, liquid, or solid), could vary dramatically over these short spatial and temporal scales. Regarding the difference in surface air pressure on Mars (<1% of that in Earth’s atmosphere),^79,80^ we note that the effect of pressure on chemical partition ratios is generally ignored by assuming the atmosphere and vapor molecules of the chemical behave like ideal gases.^74^ With the much lower atmospheric pressure on Mars, an even more ideal gas-like behavior would be expected than on Earth, so the differences in pressure would likely not have an impact on the chemical partitioning behavior.

The much drier conditions on Mars compared to those on Earth could also drive differences in chemical partitioning behavior. Subsaturation humidity levels on Earth can result in thin films of liquid water that block chemical binding sites on inorganic surfaces, with chemical adsorption exhibiting linear dependencies on relative humidity (RH) between 30 and 90% (see Chapter 11 of ref 74). Under the very dry conditions at the Martian surface (typical daily maximum of 5–15% RH measured by the Perseverance Rover),^78^ inorganic surfaces like dust particles would likely be more heterogeneous due to the lower amount of water molecules (if any) covering binding sites, further complicating the calculation of chemical partition ratios relative to current frameworks used on Earth.

Additionally, while methods have been developed to determine chemical partition ratios between air and H_2_O-based snow and ice,^81–83^ to the best of the authors’ knowledge, there have been no studies investigating how chemicals partition between air and the CO_2_ ice that covers much of the polar regions during hemispheric winters on Mars.^84,85^ Thus, there is a need to conduct a comprehensive review of available chemical dynamics equations for chemical partitioning behavior on Mars, rederivation of governing equations where possible to support application to Martian conditions, and experimental studies for chemical partitioning between relevant phases to support these theoretical approaches.

### 3.3 Persistence and Degradation

On Earth, the degradation of chemicals in the environment is driven largely by biodegradation, hydrolysis, redox reactions, direct photolysis, and indirect photolysis (e.g., hydroxyl radicals).^74^ The degradation of chemicals on Earth typically happens in a series of steps, whereby a parent chemical is converted into degradation products, which then themselves typically undergo additional degradation steps via one or more pathways, with microbe-based biodegradation often playing a role in one or more of these steps.^74^ On Mars, due to the lack of known biological activity or liquid water on the surface, biodegradation and hydrolysis will not play a major role in chemical degradation at the surface, and potential microbial or hydrolysis degradation in the subsurface can only be speculated about at this time. Thus, the degradation of chemicals on Mars would likely proceed via photolysis or reactive chemical species (e.g., hydroxyl radicals), until either a degradation product that is perfectly persistent (in the Martian environment) is generated or complete mineralization of the compound takes place (see Figure 3c). Thus, a key question in assessing how anthropogenic chemicals may pollute Mars is understanding what the terminal degradation products of anthropogenically introduced organic molecules are in the Martian environment and what route compounds go through to achieve this state.

Compared to conditions on Earth’s surface, chemicals on Mars would receive roughly the same amount of UVA radiation (315–400 nm), but approximately ten times higher levels of high energy UVB + UVC radiation (361 kJ/m^2^ of 200–315 nm radiation)^86^ due to the much lower levels of oxygen and ozone in the Martian atmosphere.^87^ Such higher amounts of UV radiation would likely cause increased rates of degradation of chemicals via direct photolysis. Furthermore, as would be the case during Earth–Mars transit, chemicals would still be exposed to galactic cosmic rays and solar energetic particles on the Martian surface.^57^ Several oxidation mechanisms have also been observed or hypothesized at the Martian surface: perchlorates, reactive oxygenated species, hydrogen peroxide, and iron-bearing species,^88^ as well as hydroxyl radicals in the atmosphere.^89,90^ However, the dynamics of such processes are not well understood. While some research has been done on the impacts of one or more of these combined degradation mechanisms on organic chemical persistence in Mars-like environments,^91–94^ results from these studies have shown that the interacting effects of organic chemicals with minerals present in Martian regolith can have either a protective or destructive effect when combined with UV degradation, depending on the organic chemical and mineral. This highlights the need for more research to understand the combined impacts of the Martian environment on the persistence of a wide range of organic chemicals.^88^

For anthropogenically introduced organic materials in the Martian environment, such as plastics, persistence will likely be determined by physical degradation (e.g., abrasion with dust^95^) as well as chemical or photochemical interactions with the environment (i.e., direct photolysis and/or interaction with reactive species).^96–98^ Some investigation has occurred into the effects of the Martian environment on materials in development for use in crewed missions to Mars. For example, Larson and Fries (2017) exposed a suite of possible extravehicular activity suit materials to vacuum and UV conditions equivalent to a 500-day surface stay on Mars and found that the chemical composition of the materials was changed due to the UV exposure, and the elongation and tensile strength of most materials decreased.^32^ The high-energy galactic cosmic rays and solar energetic particle radiation that materials would be exposed to can also change the molecular structure of materials, potentially impacting their degradation.^54^ For particles generated from the organic materials, additional degradation via the cleavage of molecules from the material (e.g., polymer) could occur. However, it is unclear whether complete mineralization would take place since, at least under Earth conditions, microbes are often responsible for the mineralization step of small molecule degradation products from plastics.^96^ Key questions regarding the persistence of organic materials in the Martian environment are thus how exposure to the environmental conditions during Earth–Mars transit may impact the molecular structure and ultimately the persistence of the material, how the Martian dust, UV and other radiation, and atmospheric conditions would affect aging and decomposition of the bulk material, and how these processes would ultimately relate to mineralization or final degradation products of the materials in the environment.

Regarding anthropogenically introduced metals and other inorganic materials, key aging processes will likely occur through UV exposure, dust abrasion, and chemical reactions with the surrounding environment. Calle (2019) assessed available information regarding the corrosion of metals used in Mars exploration activities.^99^ They found that while there is evidence that, for a common aerospace material (aerospace aluminum alloy), the small amount of oxygen in the Martian atmosphere would be able to react with it when the metal becomes scratched, additional research is needed to understand how metals would interact with brines in the Martian environment and the impact of radiation and regolith on these interactions. This was further explored by Martín-Torres et al. (2021) who found that brines, in combination with mechanical wear, would accelerate the corrosion of a material used for the wheels of the ExoMars 2020 rover (Sandvik 11R51 stainless steel).^100^ Suman and Zanini (2024) assessed the impact of erosive properties of a Martian dust simulant, compared to dust from Earth, on three materials used in spacecraft Mars missions (titanium, aluminum, and stainless steel), with impact speeds reflective of Martian wind speeds/dust devils (note, however, that experiments were conducted at ambient room temperature conditions, not reflective of Mars).^101^ They found that, depending on the material, dust loading, wind speed, and impact angle, Martian dust can cause up to 1.5 times more erosion (by weight) compared to Earth dust and that erosion with Martian dust caused increased surface roughness, compared to Earth-based dust, which generally decreased the surface roughness of the materials. Sengupta et al. (2011) also investigated the degradation of paint coatings for surface Mars missions due to retro-rocket propulsion used during the landing sequence, finding that a “space-rated” mineral-oxide paint on aluminum (zinc oxide primer with silica-filled organic overcoat) could experience pitting erosion due to the impinging of dust, silt, or sand.^65^ How these aging processes impact the production of micro- and nanoparticles released into the environment and the environmental behavior/persistence of these particles once released are key questions.

The location of anthropogenically introduced chemicals and material particles (whether organic or inorganic) in the environment could impact the potential degradation routes available and may contribute to whether complete mineralization of the contamination would occur. For example, if molecules of a chemical or particles of a material are deposited onto the surface and buried under dust or ice, this contamination would not be exposed to the same level (or any level) of photolysis or hydroxyl radicals, thus likely reducing its environmental degradation rate and increasing its persistence. Furthermore, the phase of the chemical as well as the media in which it is located (e.g., volatilized in air versus sorbed to dust) could impact photolytic degradation rates.^74^

### 3.4 Transport

For chemical contamination on Mars, the media in which the chemical is present would drive potential transport mechanisms (Figure 3d). Dust devils occur frequently on Mars,^102^ and regional dust storms are also a common feature and exhibit distinct seasonal and spatial patterns.^103^ On a multiannual basis (roughly every three years), dust storms grow to engulf the entire planet, sometimes obscuring the surface for months.^104^ Dust storms and orographic mixing can loft dust tens of kilometers into the Martian atmosphere, subjecting it to long-range transport via planetary-scale atmospheric circulation patterns.^105,106^ Anthropogenically introduced chemicals that partition to dust particles could thus be subjected to local-, regional-, and planetary-scale transport processes. Volatilized chemicals (i.e., those in air) that are transported to cloud level could partition between air and the cloud particles, subjecting the chemical to cloud-based atmospheric transport, with the chemical either revolatilizing or sorbing to the cloud nuclei upon sublimation of the water or CO_2_ in the cloud particles.^107^ Airborne chemicals that are transported to polar regions, where permanent water ice or semipermanent CO_2_ ice is present at the surface, could potentially partition to these media.^84,85,108^ Similarly, dust-sorbed chemicals could be deposited onto surface water ice or CO_2_ ice through dust deposition.^109^

For particles of anthropogenically introduced materials, similar local-, regional-, or global-scale transport processes could apply as those for chemicals. On Earth, the shape and size of microparticles are key in determining their potential for long-range transport, with fibers of microplastics undergoing longer-range transport than spherical pieces due to their slower settling rate.^73^ The shape of microplastic particles has also been found to impact transport within dust storms.^110^ Given the occurrence of planetary-scale dust storms on Mars that enable long-range transport of dust,^104^ it is unclear whether the shape and size characteristics of anthropogenically introduced micro- or nanoparticles would play a determining role in their planetary-scale transport, or whether such differential characteristics could influence the preferential accumulation of particles in certain areas. The roughly one-third gravity on Mars compared to Earth and the much lower (<1%) atmospheric density at the surface would also impact anthropogenically introduced particle transport.^79,80^ Thus, a key unknown is what characteristics (if any) of particles would make them more or less conducive to long-range transport or preferential accumulation in certain regions of the Martian environment. Such information could inform the design of materials so that, if micro- or nanoparticles are generated from them, the shapes and sizes of such particles are less likely to be transported over long distances and/or preferentially accumulate in a given medium.

The possible transport of microbes from places of human exploration to “Special Regions” of Mars has been highlighted as a key concern and coverage gap in the COSPAR regulations.^72,111^ “Special Regions” are defined as areas that have a high potential to harbor extant life or where microbial contamination introduced from Earth could flourish. While no Special Region with a high potential to harbor extant life has been unambiguously identified on Mars,^9^ several areas of Mars are hypothesized to have conditions that could be favorable for life (e.g., caves, saturated brines, and regions with ice and possible transient liquid water).^29^ The transport of anthropogenically introduced chemicals and material particles from their emission points to these areas could be a cause for concern.

One key potential difference between microbial contamination and chemical contamination is that the physicochemical properties of chemicals can make them more prone to accumulate in certain media in the environment and in certain regions based on environmental conditions. For example, on Earth, per- and polyfluoroalkyl substances (PFAS) preferentially accumulate in water bodies^21,22^ and have even been observed to have significant enrichment within sea ice brines relative to background ocean levels.^112^ This is a particular concern when considering the potential habitability of ice cap brines on Mars.^113^ Certain regions of the oceans also preferentially accumulate plastic debris, whereby the ocean’s general circulation traps floating plastic debris in synoptic-scale gyres.^114^

Preferential accumulation of chemicals in Earth’s environment can also occur whereby volatilized compounds exhibit an effective “distillation” of compounds by latitude, with lower-volatility chemicals depositing preferentially at higher latitudes.^115^ The large diurnal temperature swings and vertical temperature gradients on Mars could make such distillation processes quite different compared to Earth, but the possibility of such preferential accumulation of chemicals in the Martian environment should be explored with modeling studies.

Another possible mechanism for preferential accumulation of chemicals and particles could be via scavenging of contamination from the atmosphere through CO_2_ “snow” precipitation in the polar regions. During the northern and southern hemisphere winters, a substantial part of the Martian atmosphere (roughly 30%) transitions from gaseous CO_2_ to a solid state,^84^ resulting in a CO_2_ ice cap covering the pole down to 50° north/south latitude.^85^ While most of this ice cap forms through the direct deposition of gaseous CO_2_, between 3 and 20% falls as CO_2_ snow precipitation (at least in the southern hemisphere).^116^ On Earth, H_2_O snow can act as an efficient scavenger of atmospheric chemicals and particle pollution (e.g., microplastics) from the atmosphere to the surface.^117,118^ However, the much smaller size of CO_2_ snow crystals compared to H_2_O snow on Earth (μm vs mm size range), as well as the differential shape (cubic/octahedral for CO_2_ versus, e.g., hexagonal dendrites for H_2_O)^119,120^ could result in less efficient chemical/particle scavenging than on Earth. Nonetheless, given that the poles are the only known places where precipitation occurs on Mars (at least at the South Pole), this could be a preferential area for chemical/particle removal from the atmosphere and surface accumulation, highlighting the need for modeling studies to constrain this potential.

## 4 Research Needs and Lessons Learned from Pollution Policy Development

Informed by the review presented in Section 3 on chemical and material fate and transport processes in the Martian environment, Table 1 summarizes the key knowledge gaps and research needs that must be addressed to enable a “No- or Low-Exposure by Design” approach to chemicals and materials for use on Mars as a framework for expanded Planetary Protection guidelines.

**Table 1:** Knowledge Gaps around Anthropogenically Introduced Chemical and Material Contamination in the Martian Environment That Need to Be Addressed to Support Expanded Planetary Protection Guidelines toward a “No- or Low-Exposure by Design” Approach

Process	Knowledge gaps	Suggested approach to filling knowledge gaps	Related knowledge gaps for microbial contamination
Emissions	How will exposure to deep-space radiation affect emissions potential of chemicals and micro- and nanoparticles from materials, as well as the characteristics of these emissions?	Exposure of mission materials to deep-space transit conditions (e.g., radiation exposure, flight in low-Earth orbit or trans-Lunar space).	Understanding how deep-space transit and surface environment affects microbiome of crew and their transit environment.^72^
		Assessment of material and chemical characteristics following these exposures, in the context of potential environmental contamination.	
	How will the Martian environment affect emissions of chemicals and micro- and nanoparticles?	Weathering tests of materials using Martian environmental analogues (e.g., UV exposure, thermal stress, dust abrasion); characterization of bulk material and chemicals and particles released.	Understanding volatilization/outgassing of organic chemicals of concern for false-positive detection of biosignatures.^44^
Chemical Partitioning	Can partition ratios measured at Earth conditions be extrapolated to Mars conditions?	Review of chemical dynamics equations, and rederivation for Martian environmental conditions as needed.	-
	How will chemicals partition between the unique media in the Martian environment, especially dust and CO_2_ ice?	Bench-scale partitioning tests using key chemicals, dust analogues, and H_2_O and CO_2_ ice under Martian temperature, pressure, and atmospheric conditions.	
	How should the extreme spatial and temporal gradients in temperature be addressed when assessing chemical partitioning?	Mars environment chamber experiments of chemical partitioning dynamics with sharp thermal gradients.	
		2-D chemical fate modeling of near-surface environment.	
Persistence	How will the radiation, oxidation, and physical conditions before and after entry into the Martian environment affect the persistence of chemical and material contamination?	Chemical and material degradation tests in Mars regolith and atmosphere analogues, representing different conditions (e.g., equatorial, polar regions).	Understanding how introduced biologically relevant organics may persist on Mars.^72^
	How would chemical and material persistence vary spatially and temporally around Mars?	Should follow exposure to deep-space conditions.	
Transport	What characteristics (if any) of micro- and nanoparticles would be more conducive to long-range transport (considering lower atmospheric density, gravity, etc., compared to Earth)?	Modeling of settling and transport dynamics of particles with different sizes, shapes, and characteristics under Martian conditions.	Understanding and modeling natural transport of microbes in the Martian environment.^72^
		Lab-scale particle settling experiments in a Martian environmental chamber. Effects of lower gravity could be simulated in parabolic flights,^121^ or extrapolated from experiments at Earth gravity and higher (e.g., using ESA’s Large Diameter Centrifuge).^122,123^	Understanding transport of organic chemicals of concern for false-positive detection of biosignatures.^44^
	What are the spatiotemporal patterns of chemical and particle transport around Mars?	Global, spatialized chemical fate and transport modeling, using data collected from experiments proposed here and observations (e.g., Mars Climate Database;^125^ ).	Could also support modeling efforts of volatile chemicals on the Moon, and the concern for accumulation of these chemicals in “permanently shadowed regions”.^124^
	Are there regions of Mars that would preferentially accumulate chemicals or micro- or nanoparticles with certain characteristics?	Simplified box-modeling^126^ or latitudinal climate zone-modeling^127^ should precede spatially- and temporally dynamic modeling (e.g., BETR-Global).^128^	

Furthermore, as noted by Hader et al. (2023) there is potential for synergies in knowledge gaps and research needs around chemical and material contamination of Mars and identified knowledge gaps associated with the current focus of Planetary Protection promulgated by COSPAR.^6,9^ For example, understanding how the microbiome is impacted by transit between Earth and Mars, and while on the surface, is key to protecting crew health and minimizing contamination,^72^ which aligns with research needs identified herein regarding the impact transit and the Martian environment might have on material properties and the knock-on effects of environmental contamination. Additionally, questions surround how microbes could be transported from emission locations associated with human exploration (such as via adherence to dust particles, which could increase survivability and transport potential),^72^ something research on transport processes of chemical and particle contamination on Mars could help constrain. Such future work could build on the detailed modeling and experimental studies that have investigated the potential for microbial contamination to dislodge from the Perseverance rover and be transported in the near-field (tens of meters) vicinity of the rover, to ensure sample collection on the Martian surface avoids contamination with terrestrial biological material.^129–131^

There is also increasing concern within the Planetary Protection community regarding organic chemical contamination which could lead to false positive biological signature identification or otherwise interfere with studies of the chemical evolution of the solar system. Some modeling studies have been conducted to explore the very near-field contamination potential of organics that could be emitted from Martian rover missions in the context of false positive signatures.^44,132^ Interestingly, the need for large-scale chemical transport modeling has been highlighted for the Moon. There are concerns that volatile chemicals introduced by human lunar exploration missions could be transported via volatilization-deposition cycles to regions of high scientific interest, such as permanently shadowed regions. This could disrupt investigations into the chemical evolution of the solar system.^124,133^

The planetary-scale chemical fate and transport modeling we advocate for on Mars would help scientists and engineers understand how to design chemicals and materials that minimize the environmental concentrations of anthropogenically introduced contamination. This would help avoid harming any possible extant life as well as address the concerns about organic chemical contamination already identified by the Planetary Protection community. Furthermore, the use of filters to capture organics from venting and leakage has been suggested as a means of mitigating organic contamination.^70^ This would help address some of the concerns around chemical and particle emissions that we discuss in this study.

Another possible area for synergies supporting the development of management strategies for anthropogenic contamination on Mars is insight from efforts to manage chemical and material pollution on Earth. Numerous anthropogenic chemicals and materials introduced into Earth’s environment (e.g., PFAS, DDT, PCBs, CFCs, and plastics) have shown that the costs of unintended consequences (either financial or environmental) can be high and may be hard or impossible to reverse.^134–138^ But, with more than 350,000 chemicals and mixtures registered on the global market,^139^ coupled with their high economic value and varying levels of regulatory ambition across nations, managing the health and environmental risks of chemicals through policy remains a challenge on Earth.^138^

Despite this, some successful chemical management policies have been developed. This includes the Stockholm Convention, which resulted in the reduction in the use and environmental contamination of several persistent organic pollutants,^140^ and the REACH Regulation, which provides European Union (EU) regulators with a better understanding of the chemicals in use on the EU market and better tools to manage identified risks.^141,142^ Furthermore, UN member states are currently negotiating an international legally binding instrument to end plastic pollution, as well as the establishment of the Global Chemicals Framework and a science-policy panel to support countries in taking action on chemicals, waste, and pollution.^143–146^ The challenges with managing chemicals and materials may not be unique to Earth, and so our knowledge of (un)­successful methods, strategies, and approaches may be informative for the development of chemical and material contamination management on Mars.

Due to insufficient data requirements in regulations, chemical and material management on Earth struggles with a lack of knowledge about the properties and effects of chemicals, which is needed to properly assess the potential hazards associated with their use.^147,148^ Furthermore, there is a lack of knowledge about how and in which products chemicals are used, in part due to claims of confidentiality.^139,149^ Full transparency throughout the value chain should be a key goal of a chemical and material management policy for Mars—i.e., full disclosure of what chemicals and materials (and their hazard profiles as such knowledge becomes available) are in spacecraft and other infrastructure sent to Mars. This will allow a better understanding of the potential contamination problem on Mars and can be used as a basis for contamination management decisions such as the identification of substitution needs.^150,151^ In the context of organic chemicals and materials, such composition data are already collected (but not made fully available to researchers) in organic inventories required by COSPAR for materials present at >1 kg in a spacecraft,^9^ reducing the additional burden that would be required within the spacecraft manufacturing value chain to accomplish this type of transparency. Furthermore, to ensure that even the best efforts are not compromised, it is recommended that Planetary Protection rules be subject to transparent and rigorous compliance monitoring to detect and encourage correction of noncompliers regarding any future new rules around anthropogenically introduced chemical and material contamination. Such transparent compliance monitoring could also benefit “soft” enforcement of microbial Planetary Protection rules, which are currently not systematically monitored at an international level.^9,152^

Despite the potential similarities in chemical and material management issues between Earth and Mars, some key differences may also exist, presenting unique challenges and also potentially unique opportunities for more robust policy. For example, the chemical and material regulatory system on Earth is fragmented, with gaps and inconsistencies, and there are few international agreements with inconsistent enforcement.^138^ One advantage of a policy on Mars would be that, if applied in the context of the Planetary Protection guidelines, all nations and companies would be working under the same guidelines from the inception of the chemical and material management policy. Additionally, chemicals are deeply embedded in our way of life on Earth, which has led to difficulties in replacing chemicals where necessary.^153^ Since the overall use of chemicals and materials for space exploration is much smaller and technologies are still being developed,^53^ replacement—where necessary and where replacements can be identified—may be easier to streamline. Furthermore, policy development on Earth has been a slow process of incorporating new knowledge into chemical regulation (e.g., criteria for endocrine-disrupting compounds took approximately 30 years to implement in the European Union).^154,155^ The “soft law” approach of the COSPAR Planetary Protection guidelines enables scientific advances and emerging issues to be (relatively) quickly addressed and implemented. Modifying the guidelines does not require major international agreements to be changed, and major spacefaring nations generally agree to follow the COSPAR guidelines and their updates—despite them being nonlegally binding guidelines for how to fulfill obligations under the Outer Space Treaty.^9,152^ ^,^ This can, however, lead to weaknesses in the guidelines’ implementation (and thus the protective capacity for off-Earth environments), exemplified by a repeated effort in the United States congress to explicitly exempt nongovernmental agencies from having to follow guidelines promulgated by COSPAR to fulfill the United States’ Outer Space Treaty obligations.^156,157^

Conflict of interest is also rampant in the development of chemical and material policies on Earth.^158^ A potentially more difficult obstacle to overcome than these financial conflicts of interest in policy development on Mars could be the fact that a large motivator for crewed missions to Mars is competition between some countries on the international stage.^159–161^ Such international competition, especially in the context of any real or perceived national security threats, could result in environmental protection being highly deprioritized.

Nearly 60 years of chemical management on Earth have provided valuable insights into more and less effective approaches to achieving successful policy. Chemical and material pollution pose a significant threat to environmental health, and taking precautionary measures is far more effective than addressing issues after they emerge. This principle holds true from environmental, technical, and economic perspectives. Failing to apply these lessons to Mars would be a missed opportunity, potentially jeopardizing the capacity of scientists to study undisturbed abiotic or biotic processes on Mars and thus breaching Article IX of the Outer Space Treaty.

## 5 Synthesis

Governments and private companies have an aggressive timeline for sending humans to Mars. A large amount of chemicals and materials would be associated with such missions, and the potential interactions between this anthropogenically introduced contamination and the Martian environment are currently poorly understood. New discoveries are frequently being made that challenge science’s understanding of Mars’ past and current habitability. These include recent discoveries suggesting active mantle plumes that could provide heat for the melting of underground water ice deposits and potential liquid water being present at the surface in the lower latitudes of Mars much more recently than previously thought.^162–164^

Our understanding of microorganisms’ ability to survive in extreme environments is also constantly evolving, with microbes being discovered that can survive exceedingly harsh temperature, radiation, moisture, and nutrient-poor conditions.^165,166^ Such unexpected discoveries underscore the importance of taking a precautionary approach to the potential contamination of the Martian environment with anthropogenically introduced chemicals and materials that could cause “harmful contamination”, and the need for expanded Planetary Protection guidelines that enforce this approach.

A “No- or Low-Exposure by Design” approach to the development of chemicals and materials for use on Mars is a precautionary approach, given our lack of knowledge of potential hazards, wherein additional hazard-based approaches to chemical and material management could be incorporated as new information in this environment becomes known. Understanding the environmental emissions, partitioning, persistence, and transport characteristics of chemical and material contamination on Mars is a key step toward the goal of developing “safe” chemicals and materials on Mars, and filling the key knowledge gaps identified in this study would help address this need. Furthermore, understanding chemical fate- and transport-related issues in the extreme environmental conditions of Mars would improve our understanding of how chemical and material pollution behaves under extreme conditions on Earth. This is becoming increasingly relevant as polluting human activities further encroach into areas such as the Arctic, Antarctic, and the deep sea.^167–169^

Robust interplanetary chemical and material management, ahead of expanded human exploration, would proactively implement the lessons learned on Earth about the consequences of poorly managing pollution, making environmental protection a foundation for humanity’s exploration of Mars. Implementing this approach requires greater transparency around the types of chemicals and materials that are used in government and private missions. It also requires the environmental chemistry and Planetary Protection communities to move forward together in addressing the knowledge gaps identified in this study. The development of chemical management policies for the Martian environment could also potentially be informed by knowledge exchange with ongoing efforts to incorporate the effects of climate change into chemical policies on Earth, given that both sets of policies must be developed with large uncertainties and limits in process understanding.^170^ Furthermore, the development of Planetary Protection guidelines for anthropogenically introduced chemicals and materials on Mars would help support refined guidelines for additional celestial bodies of scientific interest that may support life in their subsurface oceans (e.g., Europa, Titan, and Enceladus), and to which exploratory missions are either underway or currently being discussed.^171–173^

## Supplementary Material



## Notes

Table S1: Estimated masses of all spacecraft that have entered Martian orbit or the Martian environment (PDF)
