# Supplementary material for: Toward “Safe” Chemicals and Materials on Mars: Knowledge Gaps for Expanding Planetary Protection Requirements
Source: Environ Sci Technol. 2026 Mar 27;60(13):9744–60. doi: 10.1021/acs.est.5c15572 (PMC13063426; doi:10.1021/acs.est.5c15572)
Supplement: Supplementary file 1 [file es5c15572_si_001.pdf]

Supporting Information for:

Toward "Safe" Chemicals and Materials on Mars: Knowledge Gaps for Expanding Planetary Protection Requirements

John D. Hader[1*], Alberto G. Fairén[2,3], Marlene Ågerstrand[4], Matthew MacLeod[4], and Bernd Nowack[1]

[1]Swiss Federal Laboratories for Materials Science and Technology (Empa), Technology and Society Laboratory, St. Gallen 9014, Switzerland.

[2]Centro de Astrobiología (CAB), CSIC-INTA, Madrid 28850, Spain.

[3]Department of Astronomy, Cornell University, Ithaca, New York, 14853, USA.

[4]Department. of Environmental Science, Stockholm University, Stockholm 106 91, Sweden.

** Email: John D. Hader, john.hader@empa.ch*

Contents:

Table S1, containing the estimated masses of all spacecraft missions sent to the Martian environment or Martian orbit (5 pages).

Table S1. Estimated masses of all spacecraft missions sent to Mars that either entered the Martian environment or Martian orbit. Primary source of mission information was the NASA Space Science Data Coordinated Archive (NSSDC).[1] Direct link to NSSDC mission data sheet provided under "Source of information", along with any additional references employed. Guidance on the list of missions obtained from The Planetary Society.[2]

Name of object	Sent by	Vehicle type	Date of arrival	Success?	Mass (kg)	Current status	Additional notes	Source of information
Mars 2-Lander	USSR	Lander	1971-11-27	Crashed	1210	Crashed	The descent module included the landing capsule, aerodynamic braking shield, a parachute system and retro-rockets; entire fuelled mass of 1210 kg.	https://nssdc.gsfc.nasa.gov/nmc/spacecraft/display.action?id=1971-045D
Mars 3-Lander	USSR	Lander	1971-12-02	Landed	1210	Defunct	(See above information provided for Mars 2-Lander)	https://nssdc.gsfc.nasa.gov/nmc/spacecraft/display.action?id=1971-049F
Mars 6-Lander	USSR	Lander	1974-03-12	Crashed	635	Crashed	Mass is the landed mass of 635 kg. No information regarding full entry mass available.	https://nssdc.gsfc.nasa.gov/nmc/spacecraft/display.action?id=1973-052A
Viking 1-Lander	USA	Lander	1976-07-20	Landed	1050	Defunct	Mass is the landing system (i.e., total launch mass minus orbiter mass).	https://nssdc.gsfc.nasa.gov/nmc/spacecraft/display.action?id=1975-075C & [3]
Viking 2-Lander	USA	Lander	1976-09-03	Landed	1050	Defunct	(See above information provided Viking 1 lander)	https://nssdc.gsfc.nasa.gov/nmc/spacecraft/display.action?id=1975-083C & [3]
Mars Pathfinder	USA	Lander & Rover	1997-07-04	Landed	801	Defunct	Mass considers launch mass (895 kg), including cruise stage, aeroshell, rover, parachute, airbags, and engines, but excludes 94 kg of cruise propellant.	https://nssdc.gsfc.nasa.gov/nmc/spacecraft/display.action?id=1996-068A & https://nssdc.gsfc.nasa.gov/nmc/spacecraft/display.action?id=MESURPR & [4]
Mars Climate Orbiter	USA	Orbiter	1999-09-23	Burned in atmosphere/ crashed	338	Crashed	Spacecraft was 338 kg. 291 kg of propellant were also on board, but for simplicity and due to lack of data, assuming all fuel was spent upon failed orbital injection burn prior to unintended atmospheric entry.	https://nssdc.gsfc.nasa.gov/nmc/spacecraft/display.action?id=1998-073A & [5]
Mars Polar Lander	USA	Lander	1999-12-03	Crashed	583	Crashed	Mass is launch mass and includes cruise stage, aeroshell, lander, and fuel. Fuel mass is included (64 kg), as landing versus cruise fuel not distinguished.	https://nssdc.gsfc.nasa.gov/nmc/spacecraft/display.action?id=1999-001A & [6] & [5]
Beagle 2	ESA	Lander	2003-12-25	Landed	69	Defunct	Mass is full packed probe, including lander, heat shield, airbags, and parachute.	https://nssdc.gsfc.nasa.gov/nmc/spacecraft/display.action?id=2003-022C & [7]
Spirit	USA	Lander & Rover	2004-01-04	Landed	827	Defunct	Reference indicates this is entry mass, and includes lander platform, heat shield, backshell, parachute, and cruise stage mass (though cruise propellant may also be included in this)	https://nssdc.gsfc.nasa.gov/nmc/spacecraft/display.action?id=2003-027A
Opportunity	USA	Lander & Rover	2004-01-25	Landed	827	Defunct	(see above notes for Spirit)	https://nssdc.gsfc.nasa.gov/nmc/spacecraft/display.action?id=2003-032A

Phoenix	USA	Lander	2008-05-25	Landed	670	Defunct	Mass is mass at launch (i.e., including cruise stage, lander, parachute, aeroshell, and propellant). Propellant mass included, since distinction between cruise versus entry fuel not made.	https://nssdc.gsfc.nasa.gov/nmc/spacecraft/display.action?id=2007-034A & [8]
Curiosity	USA	Rover	2012-08-06	Landed	3811	Operational	Mass based on re-analysis study of Curiosity entry, descent, and landing, which indicated the total wet mass of spacecraft on Mars approach as of July 29th was 3811.2 kg (landing on August 6th).	https://nssdc.gsfc.nasa.gov/nmc/spacecraft/display.action?id=2011-070A & [9] & [10]
ExoMars Schiaparelli	ESA	Lander	2016-10-19	Crashed	600	Crashed	From reference, total mass taken to include the lander, heat shield, parachute, and engines (and presumably fuel).	https://nssdc.gsfc.nasa.gov/nmc/spacecraft/display.action?id=2016-017A
InSight	USA	Lander	2018-11-26	Landed	626	Defunct	Mass includes lander (358 kg), aeroshell (189 kg), and cruise stage (79 kg). Fuel mass (67 kg) omitted to more closely match reported entry mass of roughly 608 kg (i.e., there is a slight discrepancy in reported numbers).	https://nssdc.gsfc.nasa.gov/nmc/spacecraft/display.action?id=2018-042A &[11]
Perseverance	USA	Rover	2021-02-18	Landed	4045	Operational	Mass is from re-analysis of the entry, descent, and landing, which indicates mass at launch was 4061 kg, starting propellant was 72.04 kg, and that there was 55.9 kg of propellant remaining at cruise stage separation.	https://nssdc.gsfc.nasa.gov/nmc/spacecraft/display.action?id=2020-052A & [12] & [13]
Zhurong	China	Lander & Rover	2021-05-14	Landed	1825	Hibernating or Defunct	Mass based on total launch mass of 5000 kg minus the mass of orbiter (3,175 kg). Rover was hibernated in May, 2022 due to a dust storm. Current status of rover unconfirmed by CNSA.	https://nssdc.gsfc.nasa.gov/nmc/spacecraft/display.action?id=2020-049A & [14] & [15]
Mariner 9	USA	Orbiter	1971-11-14	Entered orbit	558.8	Defunct	Mass is dry mass. Mission ended in 1972. Will enter atmosphere some time after 2022.	https://nssdc.gsfc.nasa.gov/nmc/spacecraft/display.action?id=1971-051A
Mars 2-Orbiter	USSR	Orbiter	1971-11-27	Entered orbit	2265	Defunct; no orbital information	Mass is likely dry mass. Mission declared completed in 1972.	https://nssdc.gsfc.nasa.gov/nmc/spacecraft/display.action?id=1971-045A
Mars 3-Orbiter	USSR	Orbiter	1971-12-02	Entered orbit	2265	Defunct; no orbital information	Mass is likely dry mass. Mission declared completed in 1972.	https://nssdc.gsfc.nasa.gov/nmc/spacecraft/display.action?id=1971-049A
Mars 5	USSR	Orbiter	1974-02-12	Entered orbit	2270	Defunct	Mass is likely dry mass. Mission ended in 1974.	https://nssdc.gsfc.nasa.gov/nmc/spacecraft/display.action?id=1973-049A
Viking 1-Orbiter	USA	Orbiter	1976-06-19	Entered orbit	883	Defunct	Mass is dry mass. Mission ended in 1980. Will enter atmosphere in or after 2019.	https://nssdc.gsfc.nasa.gov/nmc/spacecraft/display.action?id=1975-075A & [16]
Viking 2-Orbiter	USA	Orbiter	1976-08-07	Entered orbit	883	Defunct	Mass is dry mass. Mission ended in 1978.	https://nssdc.gsfc.nasa.gov/nmc/spacecraft/display.action?id=1975-083A
Phobos 2	USSR	Orbiter	1988-07-12 (launched)	Entered orbit	2600	Defunct	Unclear whether mass is dry or wet mass. Mission ended in 1989.	https://nssdc.gsfc.nasa.gov/nmc/spacecraft/display.action?id=1988-059A

Mars Global Surveyor	USA	Orbiter	1997-09-12	Entered orbit	1030.5	Defunct	Unclear whether mass is dry or wet mass. Mission ended in 2006.	https://nssdc.gsfc.nasa.gov/nmc/spacecraft/display.action?id=1996-062A
Mars Odyssey	USA	Orbiter	2001-10-24	Entered orbit	376.3	Operational	Mass is dry mass.	https://nssdc.gsfc.nasa.gov/nmc/spacecraft/display.action?id=2001-013A
Mars Express	ESA	Orbiter	2003-12-25	Entered orbit	666	Operational	Mass is dry mass.	https://nssdc.gsfc.nasa.gov/nmc/spacecraft/display.action?id=2003-022A
Mars Reconnaissance Orbiter	USA	Orbiter	2006-03-10	Entered orbit	1031	Operational	Mass is dry mass.	https://nssdc.gsfc.nasa.gov/nmc/spacecraft/display.action?id=2005-029A
MAVEN	USA	Orbiter	2014-09-22	Entered orbit	809	Operational	Mass is dry mass.	https://nssdc.gsfc.nasa.gov/nmc/spacecraft/display.action?id=2013-063A & [17]
Mangalyaan	India	Orbiter	2014-09-24	Entered orbit	488	Defunct	Mass is dry mass. Mission ended in 2022.	https://nssdc.gsfc.nasa.gov/nmc/spacecraft/display.action?id=2013-060A
ExoMars Trace Gas Orbiter	ESA	Orbiter	2016-10-19	Entered orbit	3732	Operational	Mass is wet mass.	https://nssdc.gsfc.nasa.gov/nmc/spacecraft/display.action?id=2016-017A
Hope Probe	UAE	Orbiter	2021-02-09	Entered orbit	550	Operational	Mass is dry mass.	https://nssdc.gsfc.nasa.gov/nmc/spacecraft/display.action?id=2020-047A
Tianwen 1	China	Orbiter	2021-02-10	Entered orbit	3175	Operational	Mass is wet mass.	https://nssdc.gsfc.nasa.gov/nmc/spacecraft/display.action?id=2020-049A & [15]

References.

(1) NASA. *NSSDC*. NASA Space Science Data Coordinated Archive. https://nssdc.gsfc.nasa.gov (accessed 2025-04-17).

(2) The Planetary Society. *Every mission to Mars ever*. Every mission to Mars ever. https://www.planetary.org/space-missions/every-mars-mission (accessed 2026-03-03).

(3) NASA. *Viking*; Press Release RELEASE NO: 75-42; 1975. https://planetary.s3.amazonaws.com/assets/presskit/viking.pdf (accessed 2026-03-03).

(4) NASA. *Mars Pathfinder Landing Press Kit*; Press Release RELEASE: 96-207; 1997. https://planetary.s3.amazonaws.com/assets/presskit/mpfland.pdf (accessed 2026-03-03).

(5) NASA. *1998 Mars Missions Press Kit*; Press Release; 1998. https://planetary.s3.amazonaws.com/assets/presskit/mars98launch.pdf (accessed 2026-03-03).

(6) JPL. Report on the Loss of the Mars Polar Lander and Deep Space 2 Missions, 2000. https://smd-cms.nasa.gov/wp-content/uploads/2023/07/3338_mpl_report_1.pdf (accessed 2026-03-03).

(7) The Open University. *technology FAQs*. Beagle2. https://web.archive.org/web/20040409174329/http:/beagle2.open.ac.uk/resources/technologyfaqs.htm (accessed 2026-03-03).

(8) NASA. *Phoenix Launch Mission to the Martian Polar North*; Press Release; 2007. https://www.jpl.nasa.gov/news/press_kits/phoenix-launch-presskit.pdf (accessed 2026-03-03).

(9) Abilleira, F. *2011 Mars Science Laboratory Trajectory Reconstruction and Performance from Launch through Landing*; AAS 04-113; NASA Jet Propulsion Laboratory, 2013. https://dataverse.jpl.nasa.gov/file.xhtml?fileId=48662&version=2.0.

(10) NASA. *Mars Science Laboratory Landing*; Press Release; 2012. https://www.jpl.nasa.gov/news/press_kits/MSLLanding.pdf (accessed 2026-03-03).

(11) NASA. *Mars InSight Landing Press Kit*; Press Release; 2018. https://planetary.s3.amazonaws.com/assets/presskit/20181031_mars_insight_landing_presskit.pdf (accessed 2026-03-03).

(12) NASA. *Mars 2020 Perseverance Launch Press Kit*; Press Release; 2020. https://www.jpl.nasa.gov/news/press_kits/mars_2020/download/mars_2020_launch_press_kit.pdf (accessed 2026-03-03).

(13) Abilleira, F.; Kruizinga, G.; et al. Mars 2020 Perseverance Trajectory Reconstruction and Performance from Launch through Landing, 2021. https://dataverse.jpl.nasa.gov/file.xhtml?fileId=65142&version=3.0 (accessed 2026-03-03).

(14) Mallapaty, S. What's Happened to China's First Mars Rover? *Nature* **2023**, d41586-023-00111–00113. https://doi.org/10.1038/d41586-023-00111-3.

(15) eoPortal. *Tianwen-1 ("Questions to Heaven"-1)*. https://www.eoportal.org/satellite-missions/tianwen-1 (accessed 2026-03-03).

(16) Jefferson, D.; Demcak, S.; Esposito, P.; Kruizinga, G. An Investigation of the Orbital Status of Viking-1. In *AIAA Guidance, Navigation, and Control Conference*; American Institute of Aeronautics and Astronautics: Chicago, Illinois, 2009. https://doi.org/10.2514/6.2009-6002.

(17) NASA. *MAVEN - NASA Science*. https://science.nasa.gov/mission/maven/ (accessed 2026-03-03).
